# Sonographic estimation of gestational age from 20 to 40 weeks by fetal kidney lengths’ measurements among pregnant women in Portharcourt, Nigeria

**DOI:** 10.1186/s12880-019-0371-z

**Published:** 2019-08-22

**Authors:** Everistus Obinna Abonyi, Charles Ugwoke Eze, Kennedy Kenechukwu Agwuna, Warric Sobechukwu Onwuzu

**Affiliations:** 1 Department of Medical Radiography and Radiological Sciences, Faculty of Health Sciences and Technology, University of Nigeria, Enugu campus, Nsukka, Enugu, Nigeria; 2 Department of Radiation Medicine, Faculty of Medicine, University of Nigeria Teaching Hospital, Ituku-Ozalla, Enugu State Nigeria

**Keywords:** Sonographic, Estimation, Kidney lengths, Gestational age

## Abstract

**Background:**

Ultrasonography has become an indispensible tool in the management of obstetric patients. Accurate determination of fetal gestational age (FGA) has posed great challenge to patient management as the accuracy of traditional biometric parameters decreases with advance in gestation age. Accuracy of fetal kidney length (FKL) in the determination of FGA at third trimester has been documented in other population. This study is aimed to create baseline reference values of fetal kidney lengths in a Nigerian population as previous studies show population specific variations.

**Methods:**

This prospective cross sectional study was carried out on 534 pregnant women between 20 and 40 weeks of gestation who met the inclusion criteria at Diamond Biomedical Services Ltd. A pilot study was conducted on 20 patients to determine the reproducibility and reliability of ultrasound measurement of FKL. Fetal kidney lengths were calculated as mean of three separate measurements. The 5th, 50th and 95th percentiles were calculated using least squared regression analysis. Third polynomial regression models were used to establish the relationship between right and left FKL and FGA.

**Results:**

Both FKL measurements are highly reproducible with excellent correlation and agreement within and between sonographers. It correlates strongly with gestational age while the relationships between right and left kidney lengths with gestational age were established using the following regression equations: *RKL* =  − 11.18 + 1.193 × *FGA* − 0.0350 × *FGA*2 + 0.00037 × *FGA*3 *and LKL* =  − 12.57 + 1.332 × *FGA* − 0.0390 × *FGA*2 + 0.00040 × *FGA*3 for right and left kidneys respectively.

**Conclusion:**

Nigerian population specific baseline reference values of FKLs for the estimation of FGA should be adopted rather than relying on the Caucasians values as universal patterns.

## Background

The importance of accurate fetal gestational age (FGA) determination in the management of obstetric patients cannot be over emphasized. The choice of obstetric management decision and its outcome depends on the knowledge of the exact age of the pregnancy. Accurate FGA determination enables adequate planning for the appropriate mode of delivery and further management of neonate after delivery [1]; helps in counseling women at risk of preterm delivery and in the evaluation of fetal growth and detection of intrauterine growth retardation (IUGR) [2]. Uncertain gestational age has been associated with adverse pregnancy outcome which includes low birth weight, spontaneous preterm delivery and perinatal mortality independence of maternal characteristics [2]. Haines [3] noted that a combination of uncertain date of LMP and any obstetric high risk situation (e.g. placenta previa, pregnancy induced hypertension, IUGR) places the fetus in jeopardy because of the difficulty in deciding the optimal time of delivery.

Different methods are being employed in the determination of FGA which includes last menstrual period (LMP), ovulation date, date of conception (in case of artificial insemination), symphysis-fundal height, quickening and ultrasonography.

Ultrasound has played a vital role in the estimation of FGA and has become an integral part of obstetric practice [4]. Sonographic estimation of gestational age is derived from calculation based on fetal measurement which serves as an indirect indicator of gestational age. Numerous equations regarding the relationship between fetal biometric parameters has been described and have proven early antenatal ultrasound to be an objective and accurate means of establishing FGA [5–8]. These biometric parameters includes- gestational sac (GS), crown rump length (CRL), biparietal diameter (BPD), head circumference (HC), abdominal circumference (AC), and femur length (FL).

Accurate assessment of FGA using ultrasound has posed a serious problem to obstetricians especially as the pregnancy approaches term. This stems from the fact that there are increased fetal biological variations as pregnancy advances. These variations can be caused by maternal age, parity, pregnancy weight, geographic location and specific population characteristics. Also technical factors like interobserver error and different measuring techniques contributes to the fetal variability as pregnancy advances to term [9]. This finding was supported by Benson and.

Doubilet [10] who noted that the accuracy of these traditional predictors of FGA (GS, CRL, BPD, HC, AC and FL) decreases as the pregnancy advances to third trimester. Gottlieb and Galan [11] therefore, suggested that in addition to these traditional parameters, ancillary biometric and non biometric measurements can help narrow the biological variability between fetuses. Butt [12] and Konje et al. [13] recommended combination of multiple biometric parameters for FGA determination in the third trimester instead of relying on a single parameter. Ansari et al. [14] and Konje et al. [13] noted that the fetal kidney length (FKL) is more accurate method of determining gestational age than the other fetal biometric indices based on BPD, HC, FL, and AC between 24 weeks and 38 weeks of gestational age.

Fetal kidney can be reliably measured using transvaginal sonography (TVS) between 14 weeks and 17 weeks of gestation while it can be measured using transabdominal ultrasonography from 18 weeks of gestation and above [15]. Study by Ansari et al. [14] shows slight variation in fetal kidney length in Asian as compared to previous studies on Caucasian. This could suggest racial variation in the studied population and may be the reason why Degan [16] stated that various epidemiological factors involved in the fetal growth should be considered and specific charts for different communities should be used when possible. His stance was corroborated by Kurtz et al. [6] who stated that there were many well established charts that have been in use for a long time but marked difference between populations sometimes necessitates researchers to build a nomogram for difference races, hence, the need for this study. There are several western studies on normal FKL but reviewed literatures have shown that no such study has been undertaken in an African descent. This study is therefore aimed to create reference range nomograms of right and left FKLs for different gestational age in a Nigerian population and also to compare the result from this study with the one done by Ansari et al. [14] on Asian population.

## Methods

This was a cross sectional study carried out at Diamond Biomedical Services Ltd., Portharcourt, Rivers state, Nigeria between February 2014 and December 2015. Simple random sampling method was used to select 534 pregnant women for the study. Patients with single fetus between 20 to 40 weeks of gestation and whose LMP agrees within 7 days with sonographically determined gestational age were included in the study. Excluded from the study were patients with twin gestation, known fetal anomaly, oligo- or poly-hydramnios and patients in labour. Patients were scanned once during the study, and to avoid double counting due to repeat scan or follow up scan, a combination of patients name and mobile phone number were used for identification. Ethical clearance was obtained from the Ethics committee of Diamond Biomedical Services Ltd. while informed consent was obtained from each patient before data collection began.

### Equipment

All sonographic examination was performed using high resolution real time digital ultrasound scanner (DP-50 by Shenzhen Mindray Biomedical Electronics Co. Ltd. China, 2011) with 3.5 MHz frequency convex transducer.

### Scanning technique

**Fig. 1:**
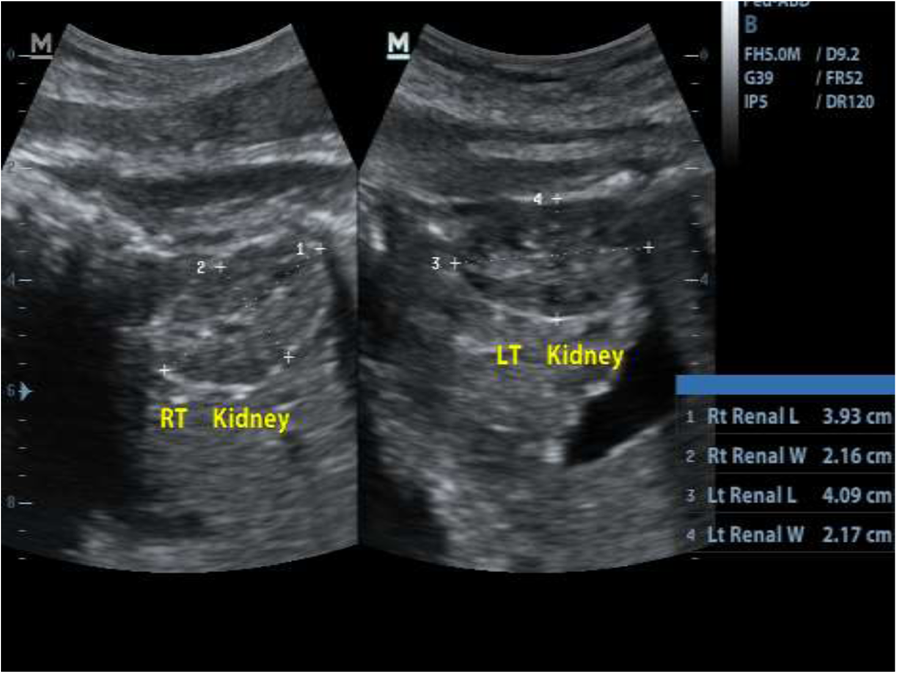
Sonogram of fetal kidney lengths measurement at 35 weeks of gestation

**A** pilot study was conducted on 20 patients to measure the reproducibility and agreement of FKL measurements within and between sonographers who have 13 years of experience each in obstetric ultrasound scan. The fetus was scanned in the transverse plane until the kidney is visualized below the stomach. The probe was then rotated through 90° to outline the longitudinal axis of the kidney. Both FKLs were measured from the outer edge of the upper pole to the outer edge of the lower pole as described by Bertagnoli et al. [17]. Care was taken so as not to include the adrenal gland in the measurement (**position for** Fig. 1). Most attempts to obtain the KFL were successful. Few that were difficult due to fetal position were obtained after few trials by gently but firmly shaking the maternal abdomen to elicit fetal movement. However, in a situation where it was not possible to get the kidney outlines well, the patient was excluded from the study.

Other measurements obtained during the study include, right and left kidney width, circumference and area. FL, BPD, HC and AC were also measured as described by Callen [18]. Three measurements of both kidney lengths and other parameters listed above were made and the average taken as the final measurement.

### Statistical analysis

Data were analyzed using R statistical package (v.2.14.12, R foundation for statistical computing, 2012) linked to SPSS v.21 (IBM Corporation, 2012).Lin’s Concordance correlation coefficient, intraclass correlation coefficient and Bland Altman plots were used to measure reproducibility and agreement of measurements within and between two sonographers.

Percentile curves were obtained using least squared regression analysis as described by Royston and Wright, 1998. The adequacy of the fitted model was tested using z- scores as well as Shapiro-Wilk’s test while QQ normality plot was used to test for consistency of the centile curves. The centile curves for the various gestational ages were calculated and the results tabulated to show the 5th, 50th and 95th percentiles. Pearson’s correlation was used to test for relation between the right and left FKLs with FL, BPD, HC and AC while line chart was used to compare the result of this study with that of Ansari et al. [14].

## Results

Out of 1002 patients who presented themselves for routine obstetric ultrasound scan within the period of study, 543 patients met the inclusion criteria and were selected for the study while 459 patients were excluded from the study for the following reasons; 345 patients were excluded for not remembering their LMP, 49 patients were excluded for the inability to assess the kidney outline, 59 patients were excluded for having fetus with IUGR, 3 patients presented with oligohydramnios while 3 patients had fetus with anomaly. The right FKL measurement ranges from 2.04 ± 0.38 cm at 20 weeks of gestation to 4.57 ± 0.26 cm at 40 weeks gestation while the left kidney measurement ranges from 2.10 ± 0.37 cm at 20 weeks of gestation to 4.75 ± 0.29 cm at 40 weeks of gestation.

### Normal ranges

**Fig. 2:**
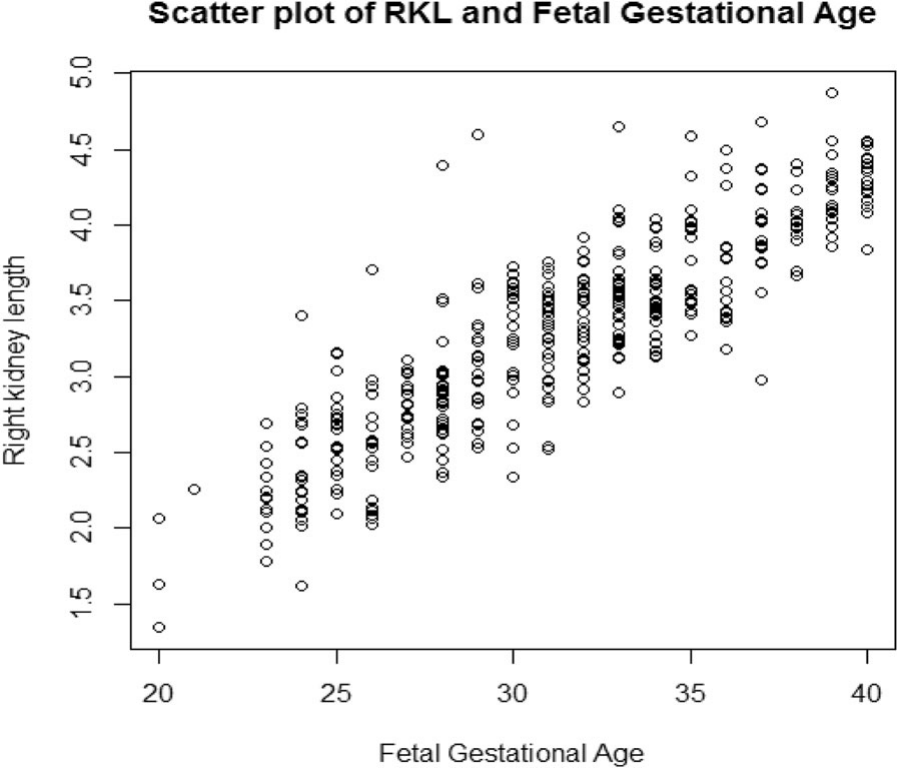
Scatter plot of Right kidney length against fetal gestational age

**Fig. 3:**
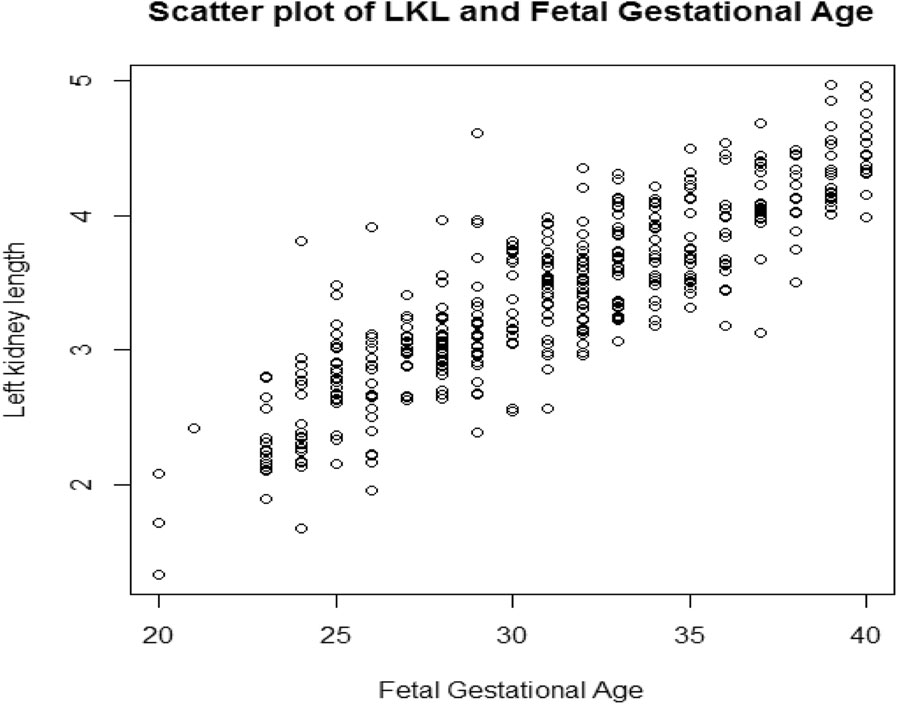
Scatter plot of Left kidney length against fetal gestational age

**Fig. 4:**
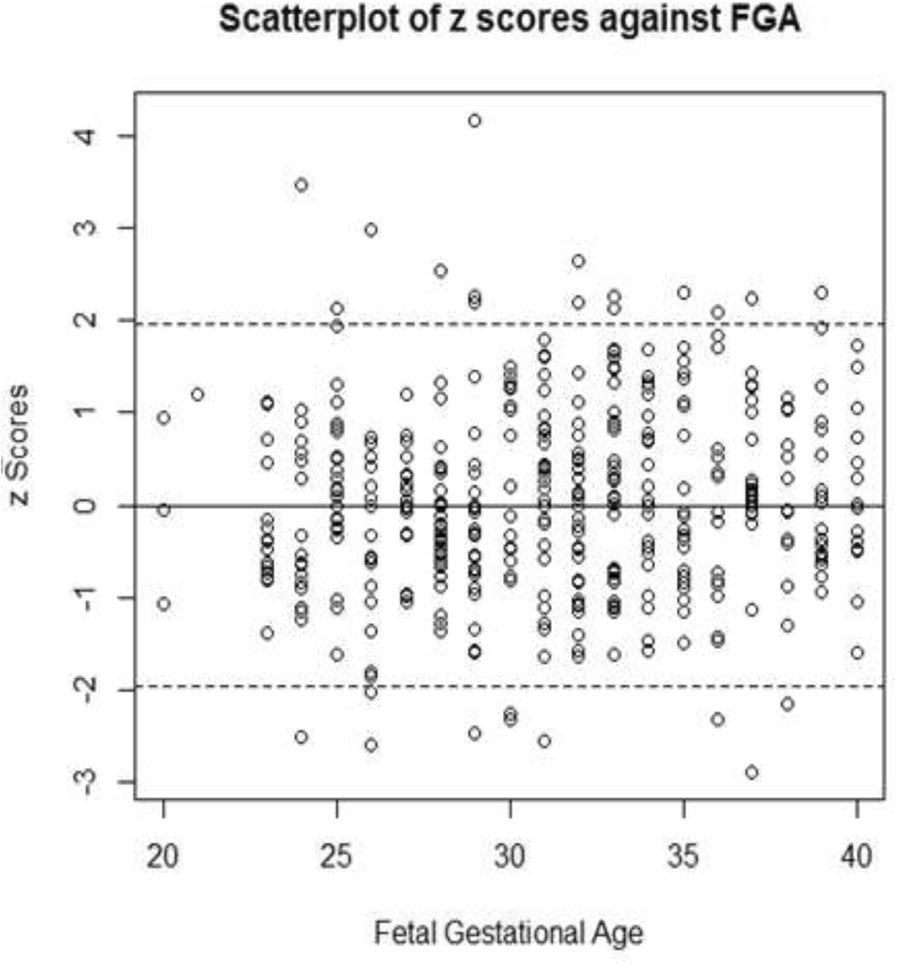
Scatter plot of z-scores against fetal gestational age

Original data was used in the analysis as log transformation increased skewness (− 0.1 to − 0.6 for the right and − 0.2 to − 0.8 for the left) (**Position for** Fig. 2 **and** Fig. 3). A third polynomial equation fitted the data for both kidneys considering the z-scores plot (**Position for** Fig. 4), and a linear equation was adequate for the SD since the plot between the scaled absolute residuals and FGA revealed no trend. The Shapiro-Wilk test retuned a value of 0.98 for the right kidney and 0.99 for the left kidney.

$$ RKL=-11.18+1.193\times FGA-0.0350\times FGA2+0.00037\times FGA3 $$

$$ SDRKL=0.5036-0.006\times FGA $$

$$ LKL=-12.57+1.332\times FGA-0.0390\times FGA2+0.00040\times FGA3 $$

$$ SDLKL=0.456-0.004\times FGA $$

The percentile chat for both kidneys is shown in Table 1. (**Position for** Table 1).

**Table 1:** Centile chart for both Kidneys Lengths

GA	Right kidney (cm)				Left kidney (cm)			
	5th	50th (Mean)	95th	SD	5th	50th (Mean)	95th	SD
20	1.03	2.04	2.29	0.38	1.11	2.10	2.34	0.37
21	1.27	2.26	2.51	0.38	1.37	2.35	2.58	0.37
23	1.67	2.64	2.87	0.37	1.80	2.76	2.99	0.36
24	1.85	2.80	3.03	0.36	1.98	2.93	3.16	0.36
25	2.00	2.94	3.16	0.35	2.15	3.08	3.31	0.35
26	2.15	3.06	3.29	0.35	2.29	3.22	3.44	0.35
27	2.28	3.18	3.40	0.34	2.43	3.34	3.56	0.34
28	2.40	3.28	3.50	0.33	2.55	3.45	3.67	0.34
29	2.51	3.38	3.59	0.33	2.66	3.55	3.77	0.34
30	2.62	3.47	3.68	0.32	2.76	3.64	3.86	0.33
31	2.72	3.56	3.76	0.32	2.86	3.73	3.94	0.33
32	2.82	3.65	3.85	0.31	2.96	3.82	4.03	0.32
33	2.93	3.73	3.93	0.30	3.06	3.91	4.11	0.32
34	3.03	3.83	4.02	0.30	3.16	4.00	4.20	0.32
35	3.15	3.92	4.11	0.29	3.27	4.10	4.30	0.31
36	3.27	4.03	4.21	0.29	3.39	4.20	4.40	0.31
37	3.40	4.14	4.32	0.28	3.51	4.32	4.51	0.30
38	3.54	4.27	4.45	0.27	3.65	4.44	4.64	0.30
39	3.70	4.41	4.58	0.27	3.81	4.59	4.78	0.30
40	3.88	4.57	4.74	0.26	3.98	4.75	4.94	0.29

### Intra rater reliability

**Fig. 5:**
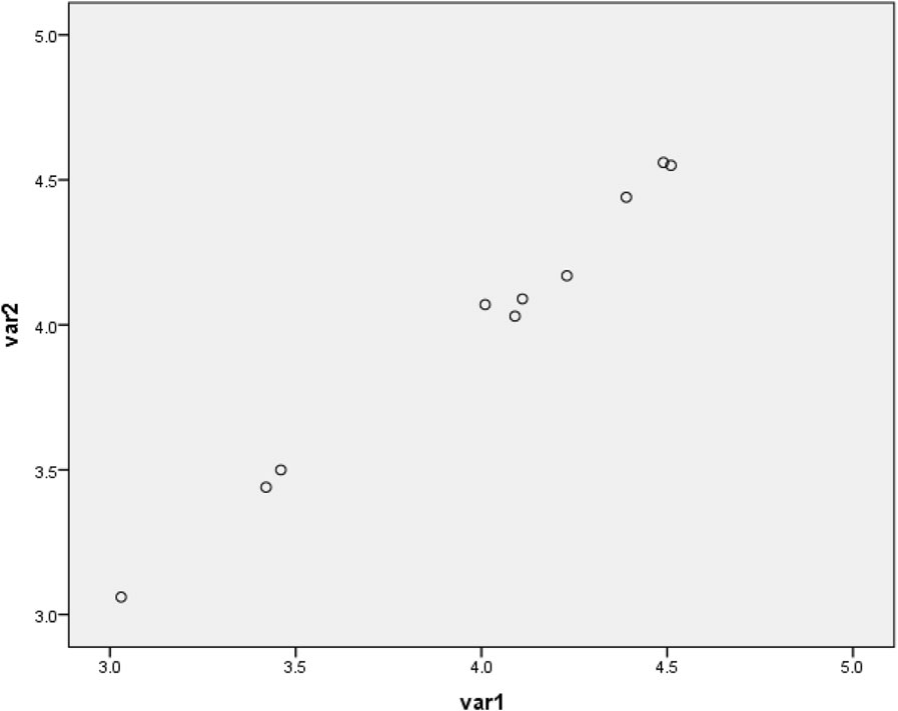
Scatter plot of Lin’s Concordance plot for a single Sonographer

**Fig. 6:**
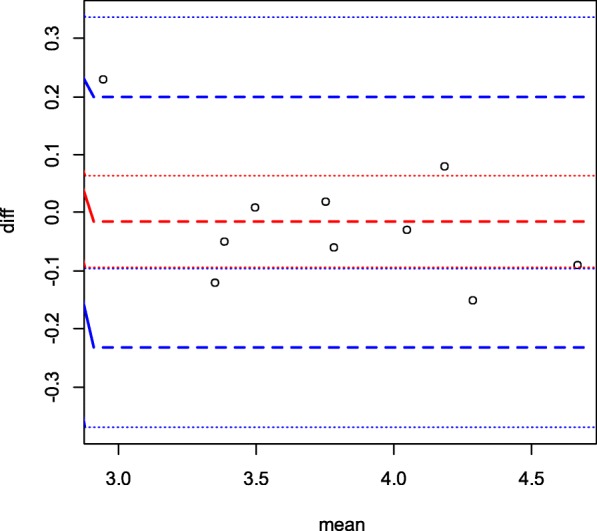
Bland Altman’s plot for measurement between two Sonographers

The Lin’s Concordance correlation coefficient was used to measure reproducibility for a single observer and a value of 0.977 was obtained which indicates excellent concordance between the two measurements. (**Position for** Fig. 5). As shown in Fig. 5, a linear relationship is demonstrated with little scatter, further indicating excellent concordance between the two measures. Using the ANOVA model, an excellent intra class correlation value of 0.989 was obtained (**Position** Table 2). Additionally, the bland Altman plot shows that the two measurements are not significantly different from zero (**position for** Fig. 6). For the two observers, a Lin’s concordance correlation gave a value of 0.997, indicating good correlation.

**Table 2:** Intraclass Correlation Coefficient for one sonographer

	Intraclass Correlation	95% Confidence Interval		F Test with True Value 0			
		Lower Bound	Upper Bound	Value	df1	df2	Sig
Single Measures	.979	.923	.995	90.759	9	9	.000
Average Measures	.989	.960	.997	90.759	9	9	.000

**Fig. 7:**
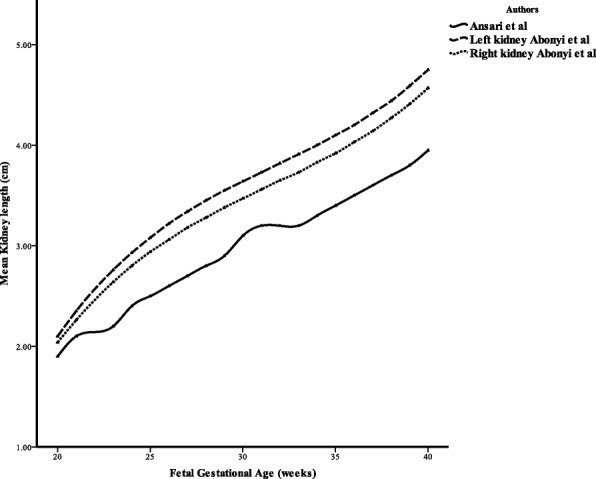
Line graph comparing our study with Ansari et al.

Furthermore, the ICC returned an excellent correlation (0.989) (**Position for** Table 3) and the Bland Altman returned a value of 0.794, which indicates good agreement. A comparison between this work and the work by Ansari et al. is shown in Fig. 7 below: (**Position for** Fig. 7).

**Table 3:** Interclass Correlation Coefficient for two sonographers

	Intraclass Correlation	95% Confidence Interval		F Test with True Value 0			
		Lower Bound	Upper Bound	Value	df1	df2	Sig
Single Measures	.979	.920	.995	87.445	9	9	.000
Average Measures	.989	.959	.997	87.445	9	9	.000

## Discussion

Effort has been on increase in finding an appropriate biometric parameter which best predicts gestational age as pregnancy advances towards term. Such biometric parameter must meet the requirement for estimating FGA which according to Campbell [19] shall have little biological variation and can be easily measured with a high degree of reproducibility and reliability. The fetal kidney can be easily visualized and measured. In the pilot study to determine inter rater reliability, the right FKL shows very excellent concordance correlation within and between sonographers with coefficient of 0.977 and 0.988 within sonographers and a coefficient value of 0.977 between sonographers while the left FKL gives a concordance coefficient of 0.995 and 0.995 within sonographers and 0.990 between sonographers. The inter class correlation coefficient is 0.996. This implies that fetal kidney measurements have excellent intra and inter rater reproducibility and reliability thus satisfying the criteria stipulated by Campbell [19]. To further confirm the finding, the intraclass correlation was calculated using the ANOVA model which gave a coefficient of 0.989, a value that is excellent enough to be used clinically. Bland Altman plot also indicated that the measurements are significantly different from zero.

From this study, both FKLs increase in a linear fashion with increase in FGA. The right FKL increases from 2.04 ± 0.38 cm at 20 weeks of gestation to 4.74 ± 0.26 cm at 40 weeks of gestation while the left FKL increases from 2.10 ± 0.37 cm at 20 weeks of gestation to 4.75 ± 0.29 cm at 40 weeks of gestation. This is in keeping with the findings of previous studies [14, 20, 21] which found that FKL increases with increase in FGA. Since FKL correlated positively with FGA, it points to its efficacy as a good predictor of FGA at third trimester. This is supported by Konje et al. [13] who stated that between 24 and 38 weeks of gestation, FKL is a more accurate method of determining FGA than the biometric indices of BPD, HC, FL and AC. This may also explain why both FKLs correlated weakly albeit positively with other biometric parameters whose efficacy decreases as gestation increases towards term. Right FKL correlates with other biometric parameters as follows: FL (0.360, *p* < 0.1), BPD (0.323, *p* < 0.01), AC (0.379, *p* < 0.01) and HC (0.311, *P* < 0.01) while the left FKL correlates as follows: FL (0.379, *p* < 0.01), BPD (0.343, *p* < 0.01), AC(0.396, *p* < 0.01) and HC (0.331, *p* < 0.01).

The measurements obtained from this study were compared with those obtained by Ansari et al. [14] and the result shows strong correlation between the two studies. This findings conforms to that of previous studies [13, 21–23] which states that though kidney size, as for all other fetal organs, is affected by growth variations, FKL remains largely unchanged as it is only the anterior-posterior and transverse diameters that are predominantly affected. Line chart was used to compare our measurements with that of Ansari et al. [14] and the result revealed that measurements from Ansari et al. [14] are always lower than that from our study. This could be attributed to the difference in the ultrasound machines used or as a result of difference in the morphology of the population studied. Also noted from this study is that left kidney is slightly longer than the right kidney which is in conformity with the study by Karim et al. [24] that found that the right kidney is shorter than the left kidney.

## Conclusion

Fetal kidney length increases with increase in FGA and shows excellent intra and inters class correlation coefficient which suggests good agreement and reproducibility of measurements. The nomograms thus produced will serve as indigenous charts rather than relying on those from Caucasians which from this study was found to be lower than that of our local population.

## Data Availability

Due to patient privacy protection, the data and corresponding materials used in this study are with the corresponding author and can only be made available upon request.

## Abbreviations

AC: Abdominal circumference
BPD: Biparietal diameter
CRL: Crown rump length
FGA: Fetal gestational age
FL: Femur length
GSD: Gestational sac diameter
HC: Head circumference
IUGR: Intrauterine growth retardation
LKL: Left kidney length
LMP: Last menstrual period
RKL: Right kidney length
